# Impulsivity and Compulsivity in Obsessive-Compulsive Spectrum Disorders: Clinical Implications for Treatment Sequencing

**DOI:** 10.7759/cureus.107663

**Published:** 2026-04-24

**Authors:** Bradley S Bohall, Alexander Gorbis, Eda Gorbis

**Affiliations:** 1 Biology, University of California, Los Angeles, Los Angeles, USA; 2 Psychology, Westwood Institute for Anxiety Disorders, Los Angeles, USA

**Keywords:** comorbidity, compulsivity, dimensional psychiatry, exposure and response prevention, impulsivity, obsessive-compulsive disorder

## Abstract

Obsessive-compulsive disorder (OCD) and related disorders comprise a heterogeneous group of neuropsychiatric conditions characterized by intrusive thoughts and repetitive behaviors. While traditionally conceptualized as disorders of compulsivity, emerging evidence supports a dimensional model in which impulsivity represents a critical and interacting construct that influences clinical presentation, comorbidity patterns, and treatment response. This interaction is increasingly understood through dysfunction within cortico-striato-thalamo-cortical circuitry and associated large-scale brain networks that regulate reward processing, habit formation, and executive control.

Comorbidity with impulsivity-related conditions, including attention-deficit/hyperactivity disorder (ADHD), body dysmorphic disorder, autism spectrum disorder, and addictive behaviors, introduces additional complexity and may significantly interfere with engagement in standard treatments such as ERP. Recent advances in neuroimaging and neurocognitive research have identified impairments in frontoparietal control networks and hub regions involved in cognitive flexibility and inhibitory control, providing a mechanistic framework for the coexistence of impulsive and compulsive features.

Pharmacological management further reflects this complexity. While selective serotonin reuptake inhibitors remain first-line treatments for compulsivity, patients with prominent impulsivity or comorbid ADHD often demonstrate an incomplete response. Emerging evidence suggests that targeted pharmacologic strategies, including stimulant and nonstimulant agents, may enhance top-down cognitive control and improve engagement with behavioral therapies when applied judiciously.

This narrative review synthesizes current neurobiological, clinical, and pharmacological evidence to examine the multidimensional relationship between impulsivity and compulsivity across OCD spectrum disorders. It proposes a sequential treatment framework in which stabilization of impulsivity and disruptive comorbidities precedes intensive OCD-focused intervention. Integrating dimensional psychiatry with neurobiological insights may improve treatment planning, enhance therapeutic engagement, and optimize outcomes in complex OCD presentations.

## Introduction and background

Obsessive-compulsive disorder (OCD) is a chronic and often debilitating psychiatric condition characterized by intrusive obsessions and repetitive compulsive behaviors that result in significant distress and functional impairment. Within the Diagnostic and Statistical Manual of Mental Disorders framework, OCD is classified among the obsessive-compulsive and related disorders (OCRDs), a group of psychiatric conditions that share core features with OCD, especially repetitive thoughts(obsessions) and/or repetitive behaviors(compulsions). Beyond OCD, this group includes body dysmorphic disorder (BDD), hoarding disorder, trichotillomania, and excoriation disorder. Although these conditions share core phenomenological features involving repetitive cognition and behavior, they demonstrate substantial heterogeneity in clinical presentation, underlying mechanisms, and treatment response [1].

Historically, OCD has been conceptualized primarily as a disorder of compulsivity, defined by repetitive, anxiety-driven behaviors performed to reduce perceived threat [1]. However, this framework is increasingly insufficient to account for the complexity observed across OCD spectrum disorders. Recent studies report high rates of comorbidity between OCD and impulsivity-related conditions, including approximately 55.9% with impulse-control disorders and up to 70% with behavioral addictions. In parallel, individuals with OCD demonstrate elevated scores on the Barratt Impulsiveness Scale and impaired performance on the Iowa Gambling Task compared with healthy controls, reflecting deficits in inhibitory control and decision-making processes consistent with increased impulsivity [2-4]. Converging evidence from neurocognitive, clinical, and neuroimaging research supports a dimensional model in which impulsivity represents a critical and interacting construct. Rather than existing as opposing phenomena, impulsivity and compulsivity are now understood as partially overlapping neurocognitive processes that may co-occur within individuals and dynamically influence symptom expression, comorbidity patterns, and therapeutic outcomes [5].

This dimensional perspective has important implications for clinical practice. A substantial proportion of individuals with OCD present with comorbid conditions involving impulsive or reward-driven behavioral processes, including attention-deficit/hyperactivity disorder (ADHD), autism spectrum disorder (ASD), BDD, and addictive behaviors. These comorbidities frequently interfere with engagement in standard first-line therapeutic treatments, such as exposure and response prevention (ERP), a specialized form of cognitive and behavioral therapy(CBT) where a therapist guides you to directly confront feared objects or situations while abstaining from the compulsive rituals you usually perform to reduce distress particularly when deficits in attention, inhibitory control, or emotional regulation are present [6]. As a result, treatment models that assume a purely compulsive pathology may fail to adequately address the mechanisms maintaining symptoms in complex presentations [7-11].

Recent advances in neuropsychiatry further support this integrative model. Dysfunction within cortico-striato-thalamo-cortical (CSTC) circuitry has long been implicated in OCD pathophysiology [12,13]; however, contemporary models emphasize the interaction of multiple parallel circuits and large-scale brain networks that regulate reward processing, habit formation, and executive control. In particular, a functional transition from ventral striatal systems associated with reward sensitivity and impulsivity to dorsal striatal systems involved in habit formation and compulsivity provides a neurobiological framework for understanding how behaviors may evolve over time. In parallel, impairments in frontoparietal control networks and disruptions in highly connected hub regions such as the anterior cingulate cortex contribute to deficits in cognitive flexibility and top-down regulation, further linking impulsive and compulsive symptom domains [14-17].

Pharmacological findings also challenge traditional assumptions regarding treatment. Selective serotonin reuptake inhibitors (SSRIs) remain the cornerstone of OCD management; however, their efficacy may be limited in individuals with significant impulsivity or comorbid ADHD [9,18]. Conversely, medications targeting dopaminergic and noradrenergic systems, including psychostimulants and nonstimulant agents, may enhance executive functioning and improve the capacity to engage in behavioral therapies when used appropriately [19,20]. These findings underscore the need for treatment strategies that address both compulsive and impulsive mechanisms rather than prioritizing one domain in isolation.

Despite growing recognition of these complexities, clinical frameworks that integrate neurobiological, pharmacological, and behavioral dimensions into practical treatment planning remain underdeveloped. In particular, the optimal sequencing of interventions in patients with co-occurring impulsive and compulsive features has not been sufficiently addressed in the literature.

The narrative review synthesizes current theoretical, neurobiological, and clinical evidence to examine the interaction between impulsivity and compulsivity across OCD spectrum disorders. It further explores the implications of this interaction for treatment planning, with a particular emphasis on a sequential treatment framework. Specifically, this review proposes that stabilizing impulsivity-related mechanisms and disruptive comorbidities before initiating intensive OCD-focused interventions may enhance treatment engagement, improve adherence, and ultimately lead to more favorable clinical outcomes.

## Review

Conceptualizing impulsivity and compulsivity

Impulsivity and compulsivity have historically been conceptualized as opposing constructs positioned along a unidimensional spectrum of behavioral control. Within this framework, impulsivity is defined as a tendency toward premature or poorly considered actions, whereas compulsivity reflects repetitive, excessive behaviors a person is compelled to perform to reduce distress or prevent a feared outcome, often leading to behavioral inhibition and rigid overcontrol. However, this dichotomous model is increasingly inadequate to explain the complexity observed across obsessive-compulsive spectrum disorders [4,21,22].

Contemporary evidence supports a dimensional framework in which impulsivity and compulsivity represent partially independent but interacting neurocognitive processes that frequently co-occur within individuals and may manifest within the same behavioral sequence. Neurocognitive research demonstrates that individuals with OCD exhibit impairments across domains associated with both constructs, including inhibitory control, cognitive flexibility, and reward processing [5,23,24]. These findings suggest that impulsivity and compulsivity are not mutually exclusive, but instead reflect overlapping dysfunction within shared cognitive and neural systems.

A key feature of this dimensional model is the dynamic transition from impulsive to compulsive behavior. Behaviors initially driven by reward sensitivity, emotional reactivity, or immediate gratification(impulsivity) may, through repetition and reinforcement, become habitual and compulsive. This progression has been observed across multiple psychiatric conditions, including trichotillomania and binge-eating disorder (BED), and parallels models of addiction in which behavioral control shifts from voluntary, reward-driven engagement to automatic, habit-based repetition [25,26].

This transition is consistent with neurobiological models describing a shift from ventral striatal systems associated with reward processing to dorsal striatal systems involved in habit formation and behavioral automation. Such findings provide a mechanistic framework for understanding how impulsive and compulsive features may coexist within the same disorder and even within the same behavior [27,28].

In addition to behavioral progression, emerging evidence suggests that impulsivity and compulsivity may represent distinct but equally significant cognitive phenotypes within OCD. A recent meta-analysis involving large clinical samples demonstrated that impairments in impulsivity and compulsivity are both present and largely independent of medication status or comorbid psychiatric conditions, supporting the conceptualization of OCD as a dual-phenotype disorder [29]. This perspective further reinforces the need to assess both dimensions during clinical evaluation rather than prioritizing one construct over the other.

From a clinical standpoint, patients may oscillate between impulsive and compulsive modes of behavior. For example, an individual may initially engage in a behavior impulsively to obtain immediate relief or gratification, but over time, the same behavior may become compulsively repeated to prevent distress or perceived harm. Consequently, accurate case formulation requires careful identification of the underlying maintaining mechanism, specifically, whether the behavior is primarily driven by reward-seeking processes, threat avoidance, or a combination of both [30,31].

This distinction has direct implications for treatment planning. Interventions that are effective for compulsive, anxiety-driven behaviors, such as ERP, a specialized form of CBT that exposes individuals to feared stimuli while preventing accompanying compulsive behaviors, may be less effective when impulsive, reward-based mechanisms predominate [6]. Conversely, strategies targeting impulsivity, including behavioral regulation (a combination of behavioral therapies to help individuals identify, pause, and modify impulsive urges before action), pharmacologic support, and interventions aimed at improving executive control, may be necessary to establish the cognitive stability required for successful engagement in OCD-specific therapies [32-34].

Recognizing the dynamic interaction between impulsivity and compulsivity provides a critical foundation for the sequential treatment framework proposed in this review. Within this framework, obsessions represent intrusive, distressing cognitive phenomena that are closely linked to dysfunctional threat processing and impaired inhibitory control. Compulsivity emerges as a maladaptive behavioral response aimed at reducing obsession-related distress, whereas impulsivity may contribute to deficits in cognitive control that increase the frequency, intrusiveness, or persistence of obsessive thoughts [35]. Accordingly, the stabilization of impulsive processes may precede and facilitate more effective intervention targeting compulsive symptomatology.

Genetic and neurobiological complexity of OCD

Understanding the biological foundations of OCD is essential for interpreting the interaction between impulsivity and compulsivity within the disorder. OCD demonstrates substantial genetic and neurobiological contributions, with twin and family studies indicating moderate heritability and higher concordance rates among monozygotic compared to dizygotic twins [36,37]. Population-based studies further support a multigenerational familial clustering pattern, reinforcing the role of inherited vulnerability in shaping the disorder’s clinical heterogeneity [37].

At the systems level, dysfunction within CSTC circuitry has long been recognized as a central feature of OCD pathophysiology [12,13]. These circuits form recurrent feedback loops linking cortical regions, particularly the orbitofrontal cortex and anterior cingulate cortex, to the striatum, globus pallidus, and thalamus, and play a critical role in regulating goal-directed behavior, emotional processing, and action selection. Within this framework, two primary pathways modulate behavioral output: the direct pathway, which facilitates action initiation through excitatory projections, and the indirect pathway, which suppresses competing or inappropriate behaviors through inhibitory control. In OCD, dysregulation of this balance, often characterized by relative hyperactivity of the direct pathway and reduced inhibitory influence of the indirect pathway, leads to thalamic disinhibition and excessive excitatory feedback to cortical regions [12,13,38]. This abnormal signaling is thought to underlie the persistence of intrusive thoughts and the inability to suppress repetitive, compulsive behaviors.

Recent neurocircuit-based models propose a five-circuit taxonomy, comprising sensorimotor, dorsal cognitive, ventral cognitive, ventral affective, and frontolimbic loops, to account for the clinical heterogeneity observed across OCD spectrum disorders. These circuits differentially modulate processes such as motor inhibition, executive functioning, reward valuation, and emotional regulation, thereby linking specific symptom profiles to underlying neurobiological dysfunction [14,15,39,40].

Within this framework, a functional distinction emerges between impulsive and compulsive behavioral processes. Impulsivity is primarily associated with ventral striatal regions, including the nucleus accumbens, which are involved in reward sensitivity and immediate reinforcement. In contrast, compulsivity is more closely linked to dorsal striatal regions, including the caudate and putamen, which mediate habit formation and the execution of repetitive, overlearned behaviors. This ventral-to-dorsal transition reflects a shift from goal-directed to habitual control and provides a neurobiological substrate for the clinical progression from impulsive engagement to compulsive repetition [14-17].

These processes are further modulated by complex neurochemical interactions, particularly involving glutamatergic and dopaminergic signaling. Within CSTC loops, the direct pathway facilitates behavior through excitatory glutamatergic projections, whereas the indirect pathway exerts inhibitory control over action selection. In OCD, dysregulation of this balance, often characterized by relative hyperactivity of the direct pathway, may reduce thalamic inhibition and contribute to the persistence of intrusive thoughts and repetitive behaviors [41-43].

Beyond localized circuit dysfunction, recent large-scale neuroimaging studies have highlighted abnormalities in distributed brain networks associated with executive control [44,45]. Meta-analytic findings demonstrate reduced activation within the frontoparietal network, including the dorsolateral prefrontal cortex and inferior parietal lobule, regions critical for cognitive flexibility and inhibitory control [16,17]. These deficits are particularly relevant to the impulsivity-compulsivity interface, as impaired top-down regulation may simultaneously increase vulnerability to impulsive responding while limiting the ability to suppress compulsive behaviors [46,47].

Importantly, these neurobiological abnormalities appear to vary across developmental stages. Evidence suggests that children with OCD exhibit distinct activation patterns compared to adults during tasks involving inhibitory control and error processing, reflecting developmental differences in neural maturation and network organization [17]. These findings support a neurodevelopmental perspective in which the relative contribution of impulsive and compulsive mechanisms may shift across the lifespan.

In addition to functional abnormalities, emerging research highlights disruptions in highly connected “hub” regions, including the anterior cingulate cortex and the putamen, which play a central role in integrating information across distributed brain networks [14,15]. These topological disruptions suggest that OCD is not solely a disorder of discrete circuits but reflects a broader impairment in network-level coordination, with implications for both cognitive control and emotional regulation.

Furthermore, neuroimaging studies indicate that effective psychotherapeutic interventions, including ERP, are associated with measurable changes in neural activity within these circuits, particularly in regions involved in emotional processing [40]. These findings underscore the plasticity of the underlying neurobiological systems and suggest that optimizing the conditions under which patients engage in therapy may enhance treatment-related neural adaptation.

Taken together, these genetic and neurobiological findings provide a mechanistic framework for understanding the coexistence of impulsivity and compulsivity within OCD spectrum disorders. The convergence of reward-processing dysfunction, habit formation, and impaired executive control helps explain the clinical heterogeneity of the disorder and reinforces the importance of treatment approaches that address both domains. In particular, deficits in top-down regulation and network integration may limit the effectiveness of standard interventions when impulsivity is not adequately stabilized, supporting the rationale for a sequential treatment framework.

Impulsivity profiles in OCD

Empirical findings indicate that impulsivity in OCD is not uniformly elevated across all domains, but instead demonstrates a differentiated and heterogeneous profile. While global trait impulsivity is not consistently increased, specific dimensions, particularly attentional impulsivity, are frequently elevated in individuals with OCD [23]. In contrast, findings related to motor impulsivity and nonplanning impulsivity remain less consistent, reflecting variability across study populations and measurement approaches [48].

This domain-specific pattern supports a dimensional framework in which subsets of individuals with OCD exhibit selective vulnerabilities to impulsive dysregulation. Attentional impulsivity, characterized by impaired sustained focus and increased distractibility, is particularly relevant in the context of comorbid neurodevelopmental conditions such as ADHD. These deficits may interfere with the cognitive demands of treatment, especially in structured interventions requiring sustained engagement, such as ERP [9,49].

Recent studies further suggest that impulsivity is not merely an associated feature but may play a central role in clinical outcomes. Evidence from large-scale analyses demonstrates that impulsivity is significantly associated with greater obsessive-compulsive symptom severity, maladaptive emotion regulation strategies, and increased risk of comorbid psychiatric conditions, including suicidality [50]. Notably, impulsivity has been identified as a mediating factor linking obsessive-compulsive symptom burden to suicidal risk, suggesting that deficits in self-regulatory capacity may contribute to adverse clinical trajectories [51].

From a neurocognitive perspective, these findings are consistent with impairments in executive control systems, particularly within frontoparietal networks responsible for attention regulation, inhibitory control, and cognitive flexibility [16,17]. Dysfunction within these systems may contribute to both increased distractibility and reduced capacity to inhibit intrusive thoughts or behavioral urges, thereby linking the impulsive and compulsive symptom domains at the level of shared cognitive deficits [52,53].

Clinically, recognizing these differentiated impulsivity profiles is essential for individualized treatment planning. Patients with prominent attentional or inhibitory control deficits may demonstrate difficulty adhering to structured therapeutic protocols, prematurely disengage from exposure exercises, or struggle to tolerate distress without resorting to avoidance or impulsive behaviors. Without targeted intervention, these impairments may limit the effectiveness of standard OCD treatments [54].

These considerations reinforce the importance of assessing impulsivity as a multidimensional construct and highlight its role in determining treatment readiness. Addressing impulsivity-related deficits may be a necessary step in optimizing engagement with OCD-specific interventions, further supporting a dimensional and sequential approach to treatment.

Comorbidity with ADHD

ADHD, a neurodevelopmental disorder characterized by a persistent pattern of inattention and/or hyperactivity-impulsivity that interferes with functioning or development, represents one of the most clinically significant comorbid conditions associated with impulsivity in OCD [1]. The co-occurrence of these disorders introduces a complex interaction between attentional dysregulation, impulsive responding, and compulsive symptomatology. Epidemiological studies estimate that ADHD is present in approximately 11%-12% of adults with OCD, with higher prevalence observed in pediatric populations [5]. Importantly, comorbid ADHD is associated with earlier onset of OCD symptoms, greater functional impairment, and poorer overall treatment outcomes [9].

From a neurobiological perspective, OCD and ADHD involve overlapping frontostriatal circuitry but are characterized by contrasting patterns of activity. OCD is typically associated with hyperactivity within these circuits, reflecting excessive error monitoring and heightened drive for control, whereas ADHD is linked to relative hypoactivity, underlying deficits in planning, attention regulation, and inhibitory control. The coexistence of these profiles may produce a clinical presentation in which individuals experience intrusive, anxiety-driven thoughts alongside diminished capacity for sustained attention and behavioral regulation [9,55].

This interaction presents a diagnostic and conceptual challenge. In some cases, attentional deficits observed in individuals with OCD may reflect “executive overload,” in which the continuous cognitive effort required to resist obsessions and suppress compulsions depletes executive resources necessary for sustained attention and organization. However, familial risk analyses support the existence of a distinct comorbid subtype, as relatives of individuals with both OCD and ADHD demonstrate increased rates of both conditions, suggesting a shared genetic vulnerability and a unique clinical phenotype characterized by earlier onset and greater severity [56].

The clinical implications of this comorbidity are substantial. ERP, the gold-standard behavioral treatment for OCD, requires sustained attention, cognitive flexibility, and the ability to tolerate distress without engaging in avoidance or impulsive responses. In individuals with comorbid ADHD, deficits in attentional control and inhibitory capacity may interfere with these processes, leading to reduced adherence, inconsistent engagement, and diminished treatment efficacy [9].

Pharmacological evidence further underscores the importance of addressing ADHD-related dysfunction in this population. Treatments targeting attentional and executive deficits, including psychostimulants and nonstimulant agents, have demonstrated reliable efficacy in improving core ADHD symptoms; however, their effects on comorbid conditions such as OCD are more variable and often require adjunctive behavioral intervention [19]. Historically, the use of stimulant medications in OCD has been approached with caution due to concerns that dopaminergic activation may exacerbate obsessions or provoke tics [57,58]. More recent evidence, however, suggests a more nuanced interaction, in which stimulant treatment may enhance top-down prefrontal control and improve the cognitive flexibility required to engage effectively in behavioral therapies [20].

These findings support a model in which ADHD-related impairments are not merely complicating factors but modifiable targets that influence treatment readiness. Stabilization of attentional capacity and impulsive control may, therefore, be a necessary precursor to effective OCD-focused intervention, particularly in patients with moderate to severe comorbid presentations.

Accordingly, a sequential treatment strategy that prioritizes managing ADHD-related dysfunction before or alongside the initiation of intensive ERP may improve treatment adherence, enhance therapeutic engagement, and ultimately lead to more favorable clinical outcomes. This perspective reinforces the broader argument that effective treatment of OCD spectrum disorders requires a dimensional approach that accounts for both impulsive and compulsive mechanisms.

Psychosis vs. overvalued ideation in OCD

Obsessive beliefs in OCD exist along a continuum of insight, ranging from intrusive doubts with preserved reality testing to fixed, delusion-like convictions. Distinguishing between psychosis and severe OCD with overvalued ideation is essential, as the underlying mechanisms and treatment approaches differ substantially [59,60].

Psychosis is a mental health condition characterized by a loss of contact with reality, disrupting a person’s thoughts, perceptions, and behaviors [61]. Psychotic beliefs are a core symptom of psychosis and are typically characterized by fixed conviction, resistance to counterevidence, and a lack of insight. Individuals experiencing these beliefs generally do not recognize them as internally generated and remain unable to revise them despite clear contradictory information. As a result, these beliefs are not amenable to cognitive or behavioral modification in the absence of pharmacologic stabilization, and treatment often requires the use of antipsychotic medications to reduce symptom severity and restore cognitive flexibility [62].

In contrast, overvalued ideation in OCD refers to strongly held beliefs that remain partially accessible to doubt. Although conviction may be high, individuals retain some degree of insight, and belief intensity may fluctuate with symptom severity. These beliefs are often responsive to cognitive restructuring, behavioral experiments, and ERP, particularly when interventions are applied in a structured and systematic manner [63,64].

The distinction between these presentations has direct implications for treatment sequencing. When beliefs are rigid and lack insight, initiating exposure-based interventions prematurely may be ineffective or poorly tolerated, as the individual is unable to engage in the cognitive processes necessary for therapeutic benefit. In such cases, pharmacologic stabilization aimed at reducing conviction and improving insight is typically required before behavioral interventions can be successfully implemented [65,66].

Conversely, when beliefs retain some degree of flexibility, early implementation of ERP and cognitive techniques may be appropriate and effective. Interventions that externalize obsessive thoughts, such as written cognitive restructuring or behavioral testing of feared outcomes, can facilitate cognitive distancing and weaken maladaptive belief systems [67,68].

Accurate assessment of insight and belief flexibility is, therefore, a critical component of clinical formulation. Differentiating psychotic processes from severe OCD with overvalued ideation ensures that treatment targets the appropriate mechanism and is delivered in the correct sequence. This distinction further reinforces the central premise of this review: that effective intervention depends not only on symptom identification, but on understanding the cognitive and neurobiological processes that maintain those symptoms [69].

Comorbidity with BDD

BDD is an obsessive-compulsive and related disorder characterized by a preoccupation with one or more perceived flaws in physical appearance that seem slight to others and performance of repetitive behaviors or mental acts in response to their perceived flaw, causing clinically significant distress or impairment in daily functioning [1]. BDD frequently co-occurs with OCD and represents one of the most clinically severe conditions within the obsessive-compulsive spectrum, a group of psychiatric and neurological conditions sharing intrusive thoughts, repetitive behaviors, and anxiety [70]. Epidemiological studies indicate that approximately 10% of individuals with primary OCD meet criteria for BDD, while a substantially higher proportion of individuals with primary BDD exhibit comorbid OCD symptoms [10]. This bidirectional overlap highlights shared underlying mechanisms involving repetitive cognition, behavioral rituals, and disturbances in self-perception [10,71].

Despite these similarities, BDD is often associated with greater clinical severity, including higher levels of functional impairment, poorer insight, and significantly increased risk of suicidality. Patients with BDD frequently present with overvalued ideation or delusional-level beliefs regarding perceived physical defects, which may be resistant to cognitive challenge and less amenable to standard behavioral interventions in the absence of stabilization [72,73].

From a dimensional perspective, BDD exemplifies the interaction between compulsive and impulsive processes. Repetitive behaviors such as mirror checking, reassurance seeking, and camouflaging may initially serve to reduce anxiety but often become rigid, habitual, and resistant to voluntary control. In more severe cases, impulsive behaviors, including cosmetic procedures or skin manipulation, may be driven by intense emotional distress and impaired inhibitory control, further complicating the clinical picture [74].

The presence of comorbid BDD has important implications for treatment planning. Elevated levels of distress, impaired insight, and increased suicidality necessitate careful assessment and prioritization of stabilization before initiating intensive exposure-based interventions. In particular, pharmacologic management and supportive interventions may be required to address mood dysregulation, reduce risk, and improve cognitive flexibility prior to engaging in ERP [75,76].

Failure to account for these factors may result in poor treatment adherence, premature dropout, or symptom exacerbation. Conversely, addressing the underlying drivers of severity, including impaired insight and emotional dysregulation, may improve readiness for OCD-specific interventions and enhance overall treatment outcomes [77].

This comorbidity further reinforces the need for a dimensional and sequential treatment approach, in which the intensity and nature of co-occurring conditions guide the timing and structure of intervention. Recognizing the distinct clinical profile of BDD within the OCD spectrum is, therefore, essential for optimizing treatment planning and reducing risk in complex presentations.

Trichotillomania: reward followed by compulsion

Trichotillomania is an obsessive-compulsive and related disorder characterized by recurrent hair pulling resulting in the loss of hair with repeated attempts to decrease or stop hair pulling, causing clinically significant distress or impairment in daily functioning [1]. Trichotillomania provides a clear clinical example of the dynamic interaction between impulsivity and compulsivity within a single disorder. Comorbidity rates between trichotillomania and OCD range from approximately 13% to 27%, reflecting overlapping neurocognitive mechanisms and shared behavioral features [25].

Hair-pulling behavior often begins as an impulsive act associated with sensory gratification, tension relief, or emotional regulation. At this stage, the behavior is typically experienced as ego-syntonic, with individuals reporting a sense of satisfaction or relief during or immediately after the act. However, with repeated engagement, the behavior becomes increasingly conditioned through negative reinforcement processes [78].

Over time, the behavior transitions from impulsive engagement to compulsive repetition. Individuals begin to experience mounting internal tension or discomfort when attempting to resist the urge to pull, and the behavior becomes more automatic, habitual, and distress-driven. At this stage, the act is often experienced as unwanted yet difficult to control, reflecting a shift toward compulsivity [79].

Clinical observations frequently describe a characteristic behavioral sequence in which an individual experiences an urge or sensory tension, engages in hair-pulling behavior to produce temporary relief, and then returns to a neutral state. This neutral state is often followed by delayed feelings of regret, shame, or distress related to the behavior. This cyclical pattern reinforces both the impulsive initiation and compulsive maintenance of the behavior [25,26].

From a neurobiological perspective, this progression parallels models of addiction in which behavioral control shifts from ventral striatal reward systems to dorsal striatal habit-based circuits. The transition reflects a shift from voluntary, reward-driven action to automatic, compulsive behavior maintained by negative reinforcement [80].

These dynamics have important implications for treatment. Interventions that target only one dimension of the behavior may be insufficient. Early-stage behaviors may respond to strategies focused on impulse control, emotional regulation, and sensory awareness, whereas later-stage compulsive behaviors may require interventions targeting habit reversal and exposure-based techniques [81].

Accordingly, effective treatment often requires an integrated approach that addresses both impulsive and compulsive mechanisms. This further supports the broader framework proposed in this review, in which identifying the dominant maintaining process and its stage of progression guides the sequencing and selection of intervention strategies.

Addictive behaviors: BED

BED is a feeding and eating disorder characterized by recurrent episodes of binge eating-defined as the consumption of an objectively large amount of food within a discrete period of time accompanied by a subjective sense of loss of control, occurring at least once weekly for three months, associated with marked psychological distress and at least three behavioral or affective features (e.g., rapid eating, eating to the point of uncomfortable fullness, eating in the absence of hunger, eating alone due to embarrassment, and subsequent feelings of disgust, depression, or guilt), and notably occurring in the absence of regular inappropriate compensatory behaviors [1]. BED represents another clinically relevant condition within the impulsive-compulsive spectrum, illustrating the transition from reward-driven behavior to compulsive repetition. Although not formally classified within OCRDs, BED shares significant neurocognitive and behavioral overlap with OCD, particularly in the domains of impaired inhibitory control, reward sensitivity, and habit formation [23,29].

In the early stages of the disorder, binge-eating behavior is often driven by impulsive mechanisms, including heightened reward sensitivity, emotional reactivity, and the pursuit of immediate gratification. Individuals may engage in binge episodes in response to stress or negative affect, with the behavior providing temporary relief or hedonic reward. At this stage, the behavior is frequently experienced as partially ego-syntonic, reinforcing its recurrence [82].

With repeated engagement, however, the behavior becomes increasingly governed by compulsive processes. Rather than being driven primarily by reward, binge-eating episodes may occur in response to internal discomfort, distress, or perceived loss of control. The behavior becomes more habitual and less consciously regulated, reflecting a shift toward negative reinforcement and compulsive repetition. Individuals often report engaging in binge-eating despite a lack of pleasure, accompanied by feelings of guilt, shame, and diminished control [83].

This progression parallels neurobiological models of addiction, in which behavioral control shifts from ventral striatal reward circuits to dorsal striatal systems involved in habit formation and automatic responding [14-17]. The convergence of impulsive and compulsive mechanisms within BED further supports the conceptualization of these constructs as interacting dimensions rather than opposing categories.

From a treatment perspective, this dual-process model has important implications. Interventions that focus exclusively on behavioral restriction or cognitive restructuring may be insufficient if underlying impulsive drivers, such as emotional dysregulation or reward sensitivity, are not addressed. Conversely, targeting impulsivity alone may fail to disrupt entrenched habitual patterns once the behavior has transitioned to a compulsive state [84,85].

Accordingly, effective management of BED often requires a staged or integrated approach that addresses both impulsive and compulsive mechanisms. Early interventions may emphasize emotional regulation, impulse control, and awareness of triggers, whereas later-stage interventions may focus on disrupting habitual patterns and modifying conditioned responses [83].

The inclusion of BED within this dimensional framework further reinforces the central premise of this review: that understanding the interaction between impulsivity and compulsivity is critical for optimizing treatment strategies across a range of psychiatric conditions. Recognizing the stage and dominant mechanism of behavior allows for more precise intervention and supports the broader application of sequential treatment models.

Comorbidity with ASD

ASD is a neurodevelopmental disorder characterized by persistent deficits in social communication and interaction, alongside restricted, repetitive patterns of behavior, interests, or activities [1]. ASD represents an important neurodevelopmental condition that frequently co-occurs with OCD at rates between 17% and 37% in youth with ASD [86,87]. This comorbidity introduces additional complexity in the clinical presentation, as repetitive behaviors and cognitive rigidity are central features of both conditions, yet may arise from distinct underlying mechanisms [88].

Individuals with ASD often exhibit restricted interests, repetitive behaviors, and intolerance of uncertainty, which can resemble compulsive symptomatology. However, unlike OCD, these behaviors are frequently experienced as ego-syntonic and may serve regulatory or self-stimulatory functions rather than being driven by intrusive thoughts or anxiety. Distinguishing between these processes is essential, as misclassification may lead to inappropriate treatment strategies [89].

Recent evidence supports the conceptualization of OCD within a broader neurodevelopmental continuum, particularly when comorbid with conditions such as ASD, ADHD, and tic disorders. In pediatric populations, individuals with comorbid neurodevelopmental disorders tend to present at a younger age, demonstrate greater functional impairment, and exhibit distinct symptom profiles, including a higher prevalence of symmetry-related behaviors and hoarding tendencies [90].

From a cognitive perspective, individuals with ASD often demonstrate deficits in cognitive flexibility, task-switching, and social cognition, which may exacerbate compulsive behaviors or interfere with adaptive coping strategies [91,92]. These impairments overlap with executive dysfunction observed in OCD, particularly within frontoparietal control networks responsible for inhibitory control and behavioral regulation [16,17]. As a result, comorbid ASD may amplify both impulsive and compulsive tendencies through shared disruptions in higher order cognitive processes [93].

The presence of ASD has significant implications for treatment. Standard ERP protocols may require modification to accommodate differences in cognitive style, learning processes, and sensory sensitivity. Individuals with ASD may benefit from more structured, concrete, and visually supported interventions, as well as increased emphasis on behavioral rehearsal and gradual skill acquisition [94].

Furthermore, deficits in emotional awareness and communication may limit engagement with traditional cognitive approaches, necessitating greater reliance on behavioral strategies and caregiver involvement. Without these adaptations, treatment adherence may be reduced, and outcomes may be suboptimal [95].

These considerations further support a dimensional and individualized approach to treatment planning. In patients with comorbid ASD, addressing underlying cognitive rigidity, sensory sensitivities, and developmental factors may be necessary before or alongside the implementation of OCD-specific interventions. This reinforces the broader framework proposed in this review, in which treatment sequencing is guided by the dominant neurocognitive and developmental features present in each case [96].

Pharmacological management

The pharmacological management of OCD, particularly in the presence of comorbid impulsivity or ADHD, presents a complex clinical challenge that reflects the underlying interaction between compulsive and impulsive neurocognitive processes. While SSRIs remain the first-line pharmacologic treatment for OCD, their efficacy is often incomplete in individuals with prominent impulsivity or executive dysfunction [97,98].

SSRIs, a class of prescription medications that increase serotonin in the brain by blocking its reuptake by presynaptic neurons, are hypothesized to exert their therapeutic effects through modulation of frontostriatal circuitry, particularly by reducing hyperactivity within CSTC loops associated with obsessive-compulsive symptomatology [12,13,99]. Although effective in reducing compulsive behaviors and intrusive thoughts, serotonergic monotherapy does not directly address deficits in attention regulation, cognitive flexibility, or inhibitory control, which are frequently present in individuals with comorbid impulsive traits. As a result, patients with co-occurring ADHD or significant attentional impairment may demonstrate only partial response to SSRI treatment and may remain functionally impaired despite reductions in obsessive-compulsive symptoms [19,100,101].

This limitation gives rise to what has been described as a pharmacological “prescribing paradox.” Historically, the use of psychostimulants such as methylphenidate in patients with OCD has been approached with caution due to concerns that increasing dopaminergic activity might exacerbate obsessive thoughts, compulsive behaviors, or tic symptoms. However, emerging evidence suggests a more nuanced interaction between dopaminergic modulation and OCD symptomatology [102,103].

Recent clinical findings indicate that stimulant medications may, in certain cases, contribute to improvement in obsessive-compulsive symptoms by enhancing top-down executive control mediated by prefrontal cortical networks [20]. By improving attention regulation and cognitive flexibility, stimulant therapy may increase a patient’s capacity to resist compulsive urges and engage more effectively in behavioral interventions such as ERP [9]. These findings are consistent with neurobiological models demonstrating that deficits in frontoparietal control networks play a central role in both impulsive and compulsive symptom domains [16,17].

Support for this approach is further strengthened by recent case series data demonstrating that methylphenidate augmentation in treatment-resistant OCD is associated with clinically meaningful reductions in symptom severity, including significant decreases in Yale-Brown Obsessive Compulsive Scale scores. In addition to symptom reduction, improvements in cognitive flexibility and treatment engagement have been observed, suggesting that stimulant therapy may enhance responsiveness to concurrent psychotherapeutic interventions [20].

In adult populations, nonstimulant pharmacologic agents such as atomoxetine and bupropion may provide additional benefit, particularly in individuals with comorbid ADHD, mood disorders, or anxiety-related conditions [19]. These agents may provide a more gradual modulation of noradrenergic and dopaminergic systems while minimizing the risk of overstimulation or symptom exacerbation in vulnerable individuals. However, their effects on obsessive-compulsive symptomatology remain variable, and they are most effective when integrated into a broader, multimodal treatment plan [104-107].

Importantly, pharmacologic interventions should not be viewed as competing approaches but rather as complementary tools that target distinct neurobiological mechanisms. SSRIs primarily address compulsive, anxiety-driven processes, whereas stimulant and nonstimulant agents target deficits in executive functioning and impulsivity. The interaction between these mechanisms underscores the need for a tailored pharmacologic management approach that accounts for the relative contribution of each dimension in individual patients [99,108].

These findings have direct implications for treatment sequencing. In patients with significant impulsivity, attentional dysregulation, or comorbid ADHD, early pharmacologic intervention targeting executive dysfunction may improve cognitive stability and enhance readiness for behavioral therapy. By strengthening top-down control processes, such interventions may create a more favorable neurocognitive environment for the successful implementation of ERP [108].

Conversely, in patients with predominantly compulsive symptomatology and preserved executive functioning, SSRIs and behavioral interventions may be initiated concurrently. This highlights the importance of individualized treatment planning guided by a dimensional understanding of symptom drivers rather than a uniform, one-size-fits-all approach [109].

Taken together, the evolving pharmacological literature supports a model in which the effective management of OCD requires simultaneous consideration of both compulsive and impulsive mechanisms. Integrating these insights into clinical practice provides a foundation for the sequential treatment framework proposed in this review, in which stabilizing impulsivity-related processes may enhance the efficacy of subsequent compulsivity-focused interventions. The major clinical and neurocognitive features of impulsivity and compulsivity across OCD spectrum conditions are summarized in Table 1.

**Table 1:** Clinical and neurocognitive profiles across the impulsivity-compulsivity spectrum in OCD-related conditions OCD: obsessive-compulsive disorder; CSTC: cortico-striato-thalamo-cortical; SSRIs: selective serotonin reuptake inhibitors; ERP: exposure and response prevention; ADHD: attention-deficit/hyperactivity disorder; BDD: body dysmorphic disorder; HRT: habit reversal training; ASD: autism spectrum disorder

Condition/comorbidity	Dominant mechanistic profile	Impulsivity features	Compulsivity features	Neurobiological basis	Treatment implications
OCD	Predominantly compulsive with impulsive overlap	Attentional impulsivity, impaired inhibitory control, poor decision-making	Rituals, avoidance behaviors, anxiety-driven repetition	CSTC dysfunction; frontoparietal control deficits	SSRIs + ERP; consider addressing executive dysfunction to optimize engagement
ADHD	Impulsivity	Inattention, disinhibition, poor executive control	Minimal primary compulsivity(secondary features possible)	Frontostriatal hypoactivity; impaired top-down control	Stabilize attention (stimulants/nonstimulants) prior to or alongside ERP to improve treatment readiness
BDD	Mixed (often severe with impaired insight)	Impulsive behaviors (e.g., cosmetic procedures, skin manipulation)	Mirror checking, reassurance seeking, rigid beliefs	Overlap with OCD circuitry; impaired insight and emotional regulation	Prioritize stabilization (insight, mood, risk) before ERP
Trichotillomania	Transition (impulsive → compulsive)	Urge-driven, reward-based initiation	Habitual, tension-reducing repetition	Ventral → dorsal striatal shift (reward → habit)	Stage-based: impulse control early, habit-focused interventions (e.g., HRT/ERP) later
Binge-eating disorder	Transition (reward → compulsive)	Reward sensitivity, emotional eating, loss of control	Compulsive binge cycles, habitual behavior	Ventral striatal reward dysfunction → dorsal habit circuits	Target emotional regulation early, then disrupt habitual patterns
ASD	Cognitive rigidity with variable impulsivity and compulsivity	Variable impulsivity; deficits in flexibility	Repetitive behaviors (often ego-syntonic)	Frontoparietal dysfunction; impaired cognitive flexibility	Modify ERP (structured, behavioral focus); address developmental factors

Treatment sequencing

The heterogeneity of OCD and its related spectrum conditions necessitates a treatment framework that extends beyond symptom-based categorization and instead accounts for the underlying neurocognitive mechanisms maintaining behavior. Traditional treatment approaches have largely emphasized the primacy of ERP alongside SSRIs. While effective for many patients, this model may be insufficient in individuals with significant impulsivity, executive dysfunction, or neurodevelopmental comorbidities [108,110].

The evidence presented throughout this review supports a dimensional framework in which impulsivity and compulsivity represent interacting but dissociable processes that differentially influence treatment readiness and response. Neurobiological findings demonstrate that impairments in frontoparietal control networks and CSTC circuitry contribute to deficits in inhibitory control, cognitive flexibility, and behavioral regulation [14-17]. These deficits may limit an individual’s capacity to engage effectively in ERP, which requires sustained attention, distress tolerance, and the ability to inhibit habitual responses [111].

Pharmacological evidence further reinforces this perspective. While SSRIs target compulsive, anxiety-driven processes, they do not directly address executive dysfunction or impulsivity [97-99]. In contrast, medications that enhance dopaminergic and noradrenergic signaling, including psychostimulants and nonstimulant agents, may improve top-down prefrontal control and increase cognitive flexibility, thereby facilitating engagement in behavioral therapy [19,20]. Clinical observations and emerging data suggest that patients with untreated attentional or inhibitory deficits may demonstrate reduced adherence to ERP protocols and diminished treatment efficacy [9].

In parallel, psychotherapeutic research highlights the importance of cognitive and behavioral readiness. Although ERP remains the gold-standard intervention, a substantial proportion of patients exhibit partial response or relapse following treatment, with emerging research identifying specific neural predictors that may account for this variability [112,113]. Trait impulsivity has been associated with greater symptom severity, poorer insight, and reduced capacity to resist compulsive urges, all of which may interfere with consistent engagement in exposure-based interventions [94,111]. Additionally, contemporary models of ERP emphasize inhibitory learning rather than habituation, requiring patients to actively form new, nonthreatening associations and tolerate uncertainty over time. These processes rely heavily on intact executive functioning and cognitive flexibility [114].

**Figure 1:**
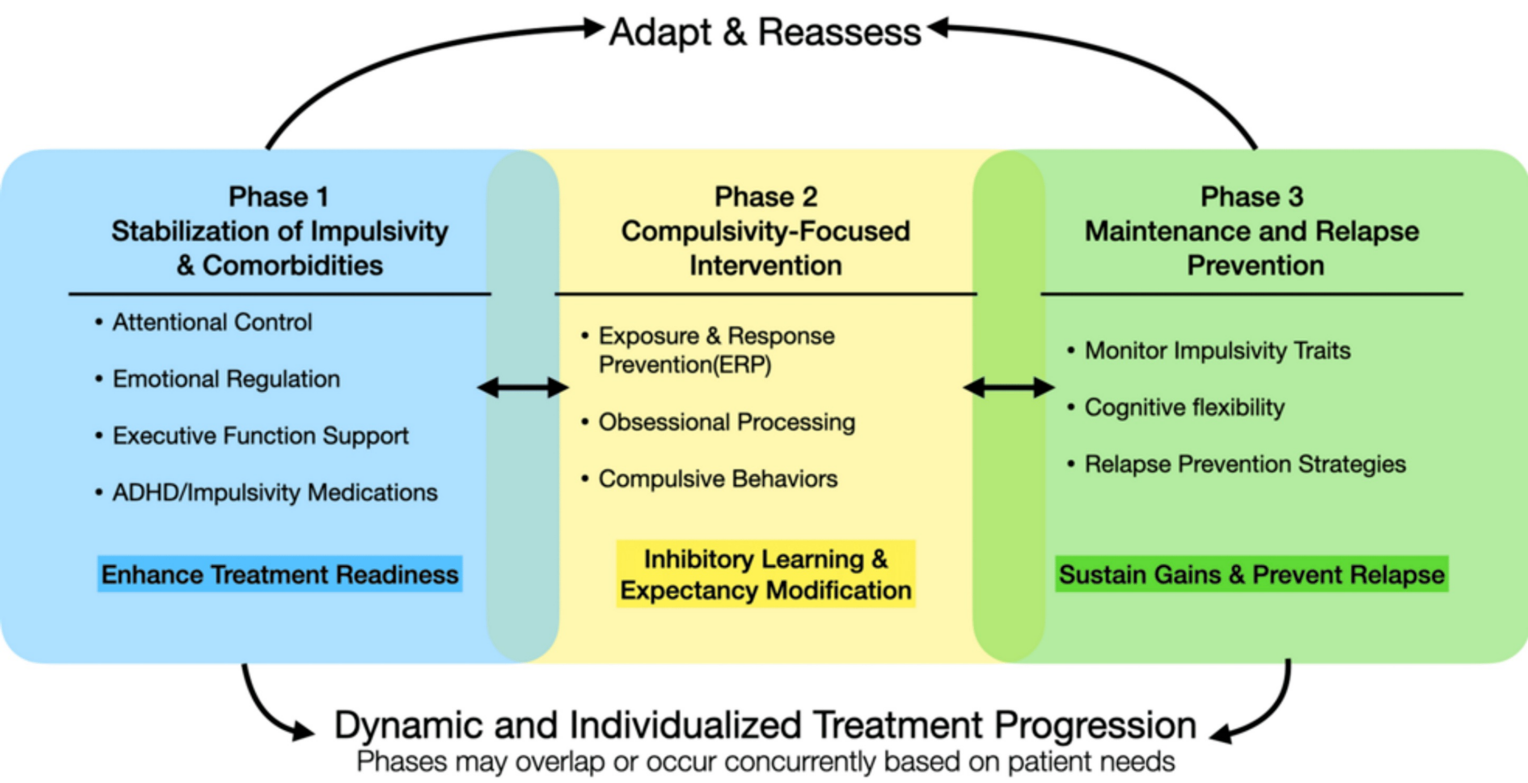
Sequential but flexible treatment framework for OCD spectrum disorders The model emphasizes initial stabilization of impulsivity-related deficits and comorbidities to enhance treatment readiness, followed by compulsivity-focused interventions such as ERP, and subsequent maintenance strategies. Bidirectional arrows and overlapping phases indicate that treatment progression is dynamic and nonlinear, with interventions adapted to individual patient needs ADHD: attention-deficit/hyperactivity disorder; ERP: exposure and response prevention; OCD: obsessive-compulsive disorder Source: This is an original image created by the authors

Taken together, these findings support a sequential treatment model in which the stabilization of impulsive processes and disruptive comorbidities precedes or occurs alongside the initiation of compulsivity-focused interventions (Figure 1).

Within this framework, obsessions act as cognitive drivers of compulsive behavior, reinforcing maladaptive threat-response associations [115]. Their effective management is achieved not through suppression, but through modification of threat expectancy and behavioral meaning. During the compulsivity-focused phase, ERP targets these processes through inhibitory learning, in which repeated exposure to obsessional cues without ritual engagement promotes the formation of nonthreatening associations and reduces the perceived necessity of compulsive responses [6,114]. These processes rely on intact cognitive flexibility and distress tolerance, further supporting the need for prior stabilization of impulsivity-related deficits to optimize therapeutic engagement [114].

Following this model, the initial phase of treatment emphasizes identifying and modulating factors that impair cognitive control, including attentional dysregulation, emotional instability, and neurodevelopmental features. Pharmacologic interventions targeting executive dysfunction, alongside behavioral strategies aimed at improving distress tolerance and self-regulation, may enhance readiness for subsequent therapeutic engagement.

Once sufficient cognitive and behavioral stability is achieved, treatment can shift toward targeted intervention for compulsive symptomatology. At this stage, ERP, particularly when implemented using principles of inhibitory learning, including expectancy violation and contextual variability, can be applied more effectively to disrupt maladaptive behavioral cycles and promote durable symptom reduction in both obsessions and compulsions [114]. In treatment-resistant cases, intensive or concentrated formats of ERP may further enhance adherence and therapeutic outcomes by reducing avoidance and increasing engagement over a shorter timeframe [116].

The final phase of treatment focuses on maintenance and relapse prevention. Given that impulsivity may persist as a trait vulnerability, ongoing monitoring of attentional control, emotional regulation, and behavioral flexibility is essential. Integrating transdiagnostic strategies that address both impulsive and compulsive tendencies may reduce the likelihood of symptom recurrence and improve long-term outcomes [111,117].

Importantly, this sequential model does not imply a rigid or linear treatment pathway. Rather, it provides a flexible, mechanism-based framework that can be adapted to the individual patient. In some cases, interventions targeting impulsivity and compulsivity may occur concurrently; however, prioritizing the stabilization of executive dysfunction in patients with significant impulsive features may enhance the overall effectiveness of subsequent treatments.

This approach represents a shift toward precision psychiatry, in which treatment is guided not solely by diagnostic categories but by the underlying neurocognitive processes that drive symptom expression. In this way, the emphasis moves from treating the disorder as a homogeneous entity to delivering individualized, patient-centered care. By aligning intervention strategies with these mechanisms, clinicians may improve treatment engagement, optimize therapeutic outcomes, and better address the complexity inherent in OCD spectrum disorders.

This framework has important implications for future research. Prospective studies are needed to evaluate the clinical utility of sequencing interventions based on neurocognitive profiles, including comparisons between sequential and concurrent treatment approaches. Further work should identify reliable predictors of treatment readiness, particularly those related to executive functioning and cognitive control, and examine the role of targeted pharmacologic augmentation in optimizing engagement with behavioral interventions. Integrating neuroimaging, behavioral, and longitudinal clinical data will be critical to validating and refining this model within precision psychiatry.

## Conclusions

The conceptualization of OCD within an impulsive-compulsive spectrum reflects a critical shift toward a more integrated understanding of psychopathology. Rather than representing opposing constructs, impulsivity and compulsivity are best understood as interacting, partially overlapping dimensions that shape symptom expression, comorbidity, and treatment response. Converging evidence from neuroimaging, neurocognitive, and clinical research highlights shared dysfunction within CSTC circuitry and frontoparietal control networks, contributing to impairments in inhibitory control, cognitive flexibility, and behavioral regulation. These overlapping mechanisms help explain the heterogeneity observed across OCD spectrum disorders and underscore the limitations of traditional models that prioritize compulsivity in isolation.

From a clinical perspective, this dimensional framework has direct implications for treatment planning. Comorbid conditions and impulsivity-related deficits frequently interfere with engagement in first-line interventions such as ERP, suggesting that treatment readiness is contingent upon underlying executive functioning and cognitive control. A sequential, mechanism-based approach, prioritizing the stabilization of impulsivity and disruptive comorbidities before or alongside compulsivity-focused interventions, offers a practical strategy to enhance therapeutic engagement and outcomes. Moving forward, continued integration of neurobiological, cognitive, and clinical data will be essential to refine this framework and advance precision psychiatry approaches that tailor interventions to the specific mechanisms driving symptomatology across the obsessive-compulsive spectrum.
